# 2,3,5-Triphenyl-2*H*-tetra­zol-3-ium tetra­phenyl­borate

**DOI:** 10.1107/S1600536812032941

**Published:** 2012-07-28

**Authors:** Hoong-Kun Fun, Tze Shyang Chia, Gamal A. E. Mostafa, Mohamed M. Hefnawy, Hatem A. Abdel-Aziz

**Affiliations:** a X-ray Crystallography Unit, School of Physics, Universiti Sains Malaysia, 11800 USM, Penang, Malaysia; b Department of Pharmaceutical Chemistry, College of Pharmacy, King Saud University, PO Box 2457, Riyadh 11451, Saudi Arabia

## Abstract

In the title salt, C_19_H_15_N_4_^+^·C_24_H_20_B^−^, the tetra­phenyl­borate anion is in a tetra­hedral geometry around the B atom [C—B—C angles of 107.10 (9)–111.79 (9)°]. In the cation, the tetra­zole ring makes dihedral angles of 3.04 (7), 51.75 (7) and 51.13 (7)° with the attached phenyl rings. In the crystal, C—H⋯π inter­actions link the cations and anions into ion pairs.

## Related literature

For applications of tetra­phenyl borate, see: Mostafa (2007 ▶); Mostafa & Al-Majed (2008 ▶); Mohamed *et al.* (2010 ▶, 2011 ▶). For the stability of the temperature controller used in the data collection, see: Cosier & Glazer (1986 ▶).
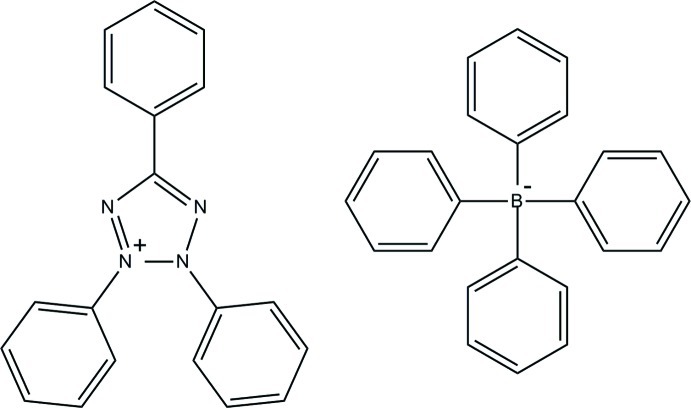


## Experimental

### 

#### Crystal data

C_19_H_15_N_4_^+^·C_24_H_20_B^−^
*M* *_r_* = 618.56
Monoclinic, 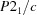

*a* = 9.8809 (1) Å
*b* = 22.6572 (3) Å
*c* = 16.0090 (2) Å
β = 110.441 (1)°
*V* = 3358.31 (7) Å^3^
*Z* = 4
Mo *K*α radiation
μ = 0.07 mm^−1^
*T* = 100 K
0.32 × 0.31 × 0.22 mm

#### Data collection

Bruker SMART APEXII CCD area-detector diffractometer
Absorption correction: multi-scan (*SADABS*; Bruker, 2009 ▶) *T*_min_ = 0.978, *T*_max_ = 0.985
37785 measured reflections
9808 independent reflections
7628 reflections with *I* > 2σ(*I*)
*R*_int_ = 0.029

#### Refinement

*R*[*F* ^2^ > 2σ(*F* ^2^)] = 0.050
*wR*(*F* ^2^) = 0.121
*S* = 1.03
9808 reflections
433 parameters
H-atom parameters constrained
Δρ_max_ = 0.33 e Å^−3^
Δρ_min_ = −0.25 e Å^−3^

### 

Data collection: *APEX2* (Bruker, 2009 ▶); cell refinement: *SAINT* (Bruker, 2009 ▶); data reduction: *SAINT*; program(s) used to solve structure: *SHELXTL* (Sheldrick, 2008 ▶); program(s) used to refine structure: *SHELXTL*; molecular graphics: *SHELXTL*; software used to prepare material for publication: *SHELXTL* and *PLATON* (Spek, 2009 ▶).

## Supplementary Material

Crystal structure: contains datablock(s) global, I. DOI: 10.1107/S1600536812032941/is5170sup1.cif

Structure factors: contains datablock(s) I. DOI: 10.1107/S1600536812032941/is5170Isup2.hkl

Additional supplementary materials:  crystallographic information; 3D view; checkCIF report

## Figures and Tables

**Table 1:** Hydrogen-bond geometry (Å, °) *Cg*1 is the centroid of the C38–C43 ring.

*D*—H⋯*A*	*D*—H	H⋯*A*	*D*⋯*A*	*D*—H⋯*A*
C10—H10*A*⋯*Cg*1^i^	0.95	2.93	3.7784 (15)	149

Symmetry code: (i) 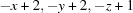.

**Fig. 1.**
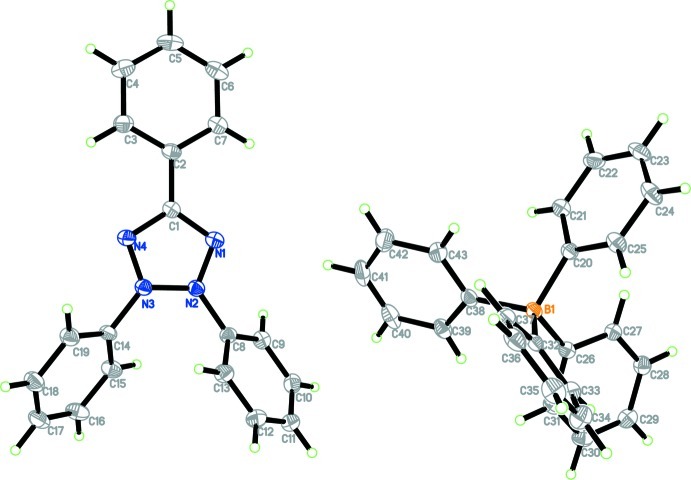
The molecular structure of the title compound with atom labels and 50% probability displacement ellipsoids.

## supplementary crystallographic information

### Comment

Tetraphenl borate reacted with triphenyltetrazolium chloride to form ion-associate complex which is used as an electroactive material for the determination of triphenyltetrazolium chloride (Mostafa, 2007; Mostafa & Al-Majed, 2008). Furthermore, tetraphenyl borate is also used as screen printing electrode (Mohamed *et al.*, 2010, 2011).

The molecular structure of the title compound is shown in Fig. 1. The asymmetric unit of the title compound, C_19_H_15_N_4_^+^.C_24_H_20_B^-^, consists of a 2,3,5-triphenyl-2*H*-tetrazol-3-ium cation and a tetraphenylborate anion. The tetraphenylborate anion is in a tetrahedral coordination geometry with B—C distances of 1.6483 (17)–1.6529 (18) Å and C—B—C tetrahedral angles of 107.10 (9)–111.79 (9)°. The dihedral angles between the phenyl rings [C20–C25 = A; C26–C31 = B; C32–C37 = C and C38–C43 = D] are A/B = 87.74 (6)°, A/C = 80.72 (6)°, A/D = 64.32 (6)°, B/C = 68.66 (6)°, B/D = 58.60 (6)° and C/D = 55.52 (7)°. In the cation, the tetrazole ring [N1–N4/C1; *r.m.s.* deviation = 0.004 Å] makes dihedral angles of 3.04 (7), 51.75 (7) and 51.13 (7)° with the attached C2–C7 (E), C8–C13 (F) and C14–C19 (G) phenyl rings, respectively. The dihedral angles between E and F rings, E and G rings, and F and G rings are 48.95 (7), 49.48 (7) and 59.49 (7)°, respectively.

In the crystal, no significant hydrogen bonds are observed, but a C—H···π interaction (Table 1) occurs, involving *Cg*1 which is the centroid of the C38–C43 ring.

### Experimental

Upon the addition of triphenyltetrazolium chloride solution (50 ml, 1×10^-2^*M*) to a solution of sodium tetraphenyl borate (50 ml), a whitish precipitate was formed. The precipitate was filtered off, washed with cold deionized water until no chloride ion was detected in the washing solution. The precipitate was dried under vacuum to give the title ion-pairs complex. Yellow blocks suitable for an X-ray structural analysis were obtained by slow evaporation from ethanol.

### Refinement

All H atoms were positioned geometrically (C—H = 0.95 Å) and refined using a riding model with *U*_iso_(H) = 1.2*U*_eq_(C). Two outliers, (5 24 8) and (5 18 5) were omitted in the final refinement.

### Crystal data

**Table:**

C_19_H_15_N_4_^+^·C_24_H_20_B^−^	*F*(000) = 1304
*M**_r_* = 618.56	*D*_x_ = 1.223 Mg m^−^^3^
Monoclinic, *P*2_1_/*c*	Mo *K*α radiation, λ = 0.71073 Å
Hall symbol: -P 2ybc	Cell parameters from 9953 reflections
*a* = 9.8809 (1) Å	θ = 2.3–29.9°
*b* = 22.6572 (3) Å	µ = 0.07 mm^−^^1^
*c* = 16.0090 (2) Å	*T* = 100 K
β = 110.441 (1)°	Block, yellow
*V* = 3358.31 (7) Å^3^	0.32 × 0.31 × 0.22 mm
*Z* = 4	

### Data collection

**Table:**

Bruker SMART APEXII CCD area-detector diffractometer	9808 independent reflections
Radiation source: fine-focus sealed tube	7628 reflections with *I* > 2σ(*I*)
Graphite monochromator	*R*_int_ = 0.029
φ and ω scans	θ_max_ = 30.1°, θ_min_ = 2.2°
Absorption correction: multi-scan (*SADABS*; Bruker, 2009)	*h* = −13→13
*T*_min_ = 0.978, *T*_max_ = 0.985	*k* = −31→15
37785 measured reflections	*l* = −19→22

### Refinement

**Table:**

Refinement on *F*^2^	Primary atom site location: structure-invariant direct methods
Least-squares matrix: full	Secondary atom site location: difference Fourier map
*R*[*F*^2^ > 2σ(*F*^2^)] = 0.050	Hydrogen site location: inferred from neighbouring sites
*wR*(*F*^2^) = 0.121	H-atom parameters constrained
*S* = 1.03	*w* = 1/[σ^2^(*F*_o_^2^) + (0.0506*P*)^2^ + 1.1876*P*] where *P* = (*F*_o_^2^ + 2*F*_c_^2^)/3
9808 reflections	(Δ/σ)_max_ = 0.001
433 parameters	Δρ_max_ = 0.33 e Å^−^^3^
0 restraints	Δρ_min_ = −0.25 e Å^−^^3^

### Special details

**Table:**

Experimental. The crystal was placed in the cold stream of an Oxford Cryosystems Cobra open-flow nitrogen cryostat (Cosier & Glazer, 1986) operating at 100.0 (1) K.
Geometry. All e.s.d.'s (except the e.s.d. in the dihedral angle between two l.s. planes) are estimated using the full covariance matrix. The cell e.s.d.'s are taken into account individually in the estimation of e.s.d.'s in distances, angles and torsion angles; correlations between e.s.d.'s in cell parameters are only used when they are defined by crystal symmetry. An approximate (isotropic) treatment of cell e.s.d.'s is used for estimating e.s.d.'s involving l.s. planes.
Refinement. Refinement of *F*^2^ against ALL reflections. The weighted *R*-factor *wR* and goodness of fit *S* are based on *F*^2^, conventional *R*-factors *R* are based on *F*, with *F* set to zero for negative *F*^2^. The threshold expression of *F*^2^ > σ(*F*^2^) is used only for calculating *R*-factors(gt) *etc*. and is not relevant to the choice of reflections for refinement. *R*-factors based on *F*^2^ are statistically about twice as large as those based on *F*, and *R*- factors based on ALL data will be even larger.

### Fractional atomic coordinates and isotropic or equivalent isotropic displacement parameters (Å^2^)

**Table:**

	*x*	*y*	*z*	*U*_iso_*/*U*_eq_	
B1	0.80855 (14)	0.91994 (6)	0.75446 (9)	0.0166 (2)	
N1	0.84759 (11)	0.86398 (5)	0.23476 (7)	0.0195 (2)	
N2	0.97900 (11)	0.84218 (4)	0.26305 (7)	0.0180 (2)	
N3	0.99069 (11)	0.80074 (4)	0.20630 (7)	0.0182 (2)	
N4	0.86703 (11)	0.79398 (5)	0.14081 (7)	0.0193 (2)	
C1	0.78000 (13)	0.83373 (5)	0.15918 (8)	0.0183 (2)	
C2	0.62910 (12)	0.84201 (5)	0.10218 (8)	0.0192 (2)	
C3	0.57066 (14)	0.80618 (6)	0.02706 (9)	0.0261 (3)	
H3A	0.6279	0.7765	0.0137	0.031*	
C4	0.42804 (15)	0.81438 (7)	−0.02773 (9)	0.0307 (3)	
H4A	0.3873	0.7902	−0.0789	0.037*	
C5	0.34501 (14)	0.85772 (7)	−0.00796 (10)	0.0309 (3)	
H5A	0.2473	0.8630	−0.0455	0.037*	
C6	0.40334 (14)	0.89346 (7)	0.06610 (10)	0.0300 (3)	
H6A	0.3458	0.9233	0.0789	0.036*	
C7	0.54578 (14)	0.88581 (6)	0.12175 (9)	0.0247 (3)	
H7A	0.5860	0.9102	0.1727	0.030*	
C8	1.09045 (12)	0.85755 (5)	0.34642 (8)	0.0185 (2)	
C9	1.12262 (13)	0.91679 (6)	0.36390 (8)	0.0212 (2)	
H9A	1.0747	0.9464	0.3221	0.025*	
C10	1.22789 (13)	0.93117 (6)	0.44512 (9)	0.0241 (3)	
H10A	1.2528	0.9713	0.4595	0.029*	
C11	1.29656 (14)	0.88727 (7)	0.50507 (9)	0.0270 (3)	
H11A	1.3683	0.8976	0.5603	0.032*	
C12	1.26162 (15)	0.82821 (7)	0.48527 (9)	0.0283 (3)	
H12A	1.3099	0.7985	0.5269	0.034*	
C13	1.15675 (14)	0.81254 (6)	0.40508 (8)	0.0228 (3)	
H13A	1.1312	0.7724	0.3908	0.027*	
C14	1.11995 (12)	0.76850 (5)	0.21228 (8)	0.0189 (2)	
C15	1.24583 (13)	0.79961 (6)	0.22342 (9)	0.0239 (3)	
H15A	1.2487	0.8414	0.2277	0.029*	
C16	1.36759 (14)	0.76719 (7)	0.22813 (9)	0.0282 (3)	
H16A	1.4558	0.7870	0.2357	0.034*	
C17	1.36125 (14)	0.70611 (6)	0.22188 (9)	0.0279 (3)	
H17A	1.4457	0.6843	0.2266	0.034*	
C18	1.23252 (14)	0.67671 (6)	0.20882 (9)	0.0273 (3)	
H18A	1.2289	0.6349	0.2034	0.033*	
C19	1.10881 (14)	0.70779 (6)	0.20366 (9)	0.0232 (3)	
H19A	1.0199	0.6881	0.1946	0.028*	
C20	0.64315 (12)	0.93994 (5)	0.74430 (8)	0.0173 (2)	
C21	0.56120 (13)	0.97844 (6)	0.67707 (8)	0.0219 (2)	
H21A	0.5987	0.9901	0.6324	0.026*	
C22	0.42720 (14)	1.00029 (6)	0.67289 (9)	0.0264 (3)	
H22A	0.3753	1.0262	0.6259	0.032*	
C23	0.36946 (13)	0.98437 (6)	0.73711 (10)	0.0272 (3)	
H23A	0.2780	0.9990	0.7345	0.033*	
C24	0.44726 (14)	0.94681 (6)	0.80501 (10)	0.0268 (3)	
H24A	0.4094	0.9358	0.8498	0.032*	
C25	0.58108 (13)	0.92496 (6)	0.80817 (9)	0.0221 (2)	
H25A	0.6321	0.8990	0.8552	0.026*	
C26	0.91901 (12)	0.97161 (5)	0.81252 (7)	0.0166 (2)	
C27	0.87021 (13)	1.02357 (5)	0.84049 (8)	0.0184 (2)	
H27A	0.7693	1.0311	0.8201	0.022*	
C28	0.96281 (13)	1.06457 (5)	0.89671 (8)	0.0209 (2)	
H28A	0.9243	1.0989	0.9141	0.025*	
C29	1.11130 (13)	1.05563 (6)	0.92752 (8)	0.0220 (2)	
H29A	1.1748	1.0830	0.9671	0.026*	
C30	1.16490 (13)	1.00586 (6)	0.89918 (8)	0.0221 (2)	
H30A	1.2662	0.9995	0.9181	0.027*	
C31	1.07066 (13)	0.96529 (5)	0.84317 (8)	0.0191 (2)	
H31A	1.1103	0.9317	0.8246	0.023*	
C32	0.84247 (12)	0.85738 (5)	0.81111 (8)	0.0168 (2)	
C33	0.93272 (13)	0.85443 (5)	0.90081 (8)	0.0200 (2)	
H33A	0.9880	0.8883	0.9272	0.024*	
C34	0.94493 (15)	0.80416 (6)	0.95301 (9)	0.0249 (3)	
H34A	1.0090	0.8041	1.0132	0.030*	
C35	0.86451 (14)	0.75428 (6)	0.91792 (9)	0.0255 (3)	
H35A	0.8699	0.7204	0.9539	0.031*	
C36	0.77569 (13)	0.75493 (5)	0.82884 (9)	0.0234 (3)	
H36A	0.7205	0.7209	0.8032	0.028*	
C37	0.76686 (13)	0.80496 (5)	0.77680 (8)	0.0210 (2)	
H37A	0.7074	0.8037	0.7156	0.025*	
C38	0.82089 (13)	0.91126 (5)	0.65512 (8)	0.0186 (2)	
C39	0.93787 (14)	0.93094 (6)	0.63216 (9)	0.0224 (2)	
H39A	1.0160	0.9499	0.6765	0.027*	
C40	0.94472 (16)	0.92393 (6)	0.54703 (9)	0.0295 (3)	
H40A	1.0262	0.9382	0.5347	0.035*	
C41	0.83374 (17)	0.89645 (6)	0.48087 (9)	0.0319 (3)	
H41A	0.8378	0.8917	0.4228	0.038*	
C42	0.71608 (16)	0.87582 (7)	0.50041 (9)	0.0312 (3)	
H42A	0.6390	0.8566	0.4556	0.037*	
C43	0.71068 (14)	0.88322 (6)	0.58572 (8)	0.0248 (3)	
H43A	0.6289	0.8687	0.5975	0.030*	

### Atomic displacement parameters (Å^2^)

**Table:**

	*U*^11^	*U*^22^	*U*^33^	*U*^12^	*U*^13^	*U*^23^
B1	0.0146 (6)	0.0176 (6)	0.0184 (6)	0.0008 (5)	0.0070 (5)	−0.0009 (5)
N1	0.0170 (5)	0.0187 (5)	0.0225 (5)	0.0009 (4)	0.0065 (4)	0.0021 (4)
N2	0.0173 (5)	0.0168 (5)	0.0199 (5)	0.0006 (4)	0.0063 (4)	0.0002 (4)
N3	0.0160 (4)	0.0182 (5)	0.0205 (5)	0.0000 (4)	0.0063 (4)	0.0000 (4)
N4	0.0159 (5)	0.0190 (5)	0.0220 (5)	0.0000 (4)	0.0052 (4)	0.0008 (4)
C1	0.0180 (5)	0.0167 (5)	0.0213 (5)	−0.0002 (4)	0.0080 (4)	0.0023 (4)
C2	0.0158 (5)	0.0194 (5)	0.0222 (6)	0.0001 (4)	0.0062 (4)	0.0042 (5)
C3	0.0217 (6)	0.0246 (6)	0.0287 (6)	0.0013 (5)	0.0047 (5)	−0.0024 (5)
C4	0.0238 (6)	0.0338 (8)	0.0276 (7)	−0.0025 (6)	0.0004 (5)	0.0000 (6)
C5	0.0173 (6)	0.0377 (8)	0.0334 (7)	0.0025 (6)	0.0035 (5)	0.0106 (6)
C6	0.0216 (6)	0.0312 (7)	0.0381 (7)	0.0076 (6)	0.0114 (6)	0.0061 (6)
C7	0.0212 (6)	0.0254 (6)	0.0278 (6)	0.0024 (5)	0.0090 (5)	0.0012 (5)
C8	0.0154 (5)	0.0211 (6)	0.0184 (5)	−0.0010 (4)	0.0053 (4)	0.0000 (4)
C9	0.0225 (6)	0.0198 (6)	0.0232 (6)	−0.0008 (5)	0.0101 (5)	0.0011 (5)
C10	0.0226 (6)	0.0248 (6)	0.0268 (6)	−0.0053 (5)	0.0111 (5)	−0.0061 (5)
C11	0.0204 (6)	0.0350 (7)	0.0234 (6)	0.0004 (5)	0.0049 (5)	−0.0063 (5)
C12	0.0271 (7)	0.0316 (7)	0.0216 (6)	0.0069 (6)	0.0028 (5)	0.0021 (5)
C13	0.0239 (6)	0.0203 (6)	0.0230 (6)	0.0010 (5)	0.0065 (5)	0.0019 (5)
C14	0.0153 (5)	0.0213 (6)	0.0199 (5)	0.0035 (4)	0.0057 (4)	0.0012 (5)
C15	0.0197 (6)	0.0217 (6)	0.0301 (6)	0.0001 (5)	0.0084 (5)	0.0039 (5)
C16	0.0175 (6)	0.0324 (7)	0.0348 (7)	0.0010 (5)	0.0094 (5)	0.0059 (6)
C17	0.0217 (6)	0.0324 (7)	0.0302 (7)	0.0089 (5)	0.0097 (5)	0.0045 (6)
C18	0.0260 (6)	0.0223 (6)	0.0331 (7)	0.0051 (5)	0.0100 (5)	0.0001 (5)
C19	0.0196 (6)	0.0209 (6)	0.0286 (6)	−0.0002 (5)	0.0077 (5)	−0.0007 (5)
C20	0.0154 (5)	0.0156 (5)	0.0207 (5)	−0.0015 (4)	0.0061 (4)	−0.0042 (4)
C21	0.0192 (6)	0.0236 (6)	0.0223 (6)	0.0022 (5)	0.0067 (5)	−0.0013 (5)
C22	0.0191 (6)	0.0250 (6)	0.0298 (7)	0.0047 (5)	0.0017 (5)	−0.0043 (5)
C23	0.0145 (5)	0.0241 (6)	0.0431 (8)	−0.0005 (5)	0.0103 (5)	−0.0100 (6)
C24	0.0224 (6)	0.0234 (6)	0.0411 (7)	−0.0021 (5)	0.0193 (6)	−0.0044 (6)
C25	0.0204 (6)	0.0195 (6)	0.0290 (6)	−0.0005 (5)	0.0121 (5)	−0.0011 (5)
C26	0.0166 (5)	0.0169 (5)	0.0174 (5)	0.0001 (4)	0.0071 (4)	0.0023 (4)
C27	0.0176 (5)	0.0189 (5)	0.0185 (5)	0.0014 (4)	0.0062 (4)	0.0003 (4)
C28	0.0238 (6)	0.0180 (5)	0.0215 (6)	0.0011 (5)	0.0087 (5)	0.0001 (5)
C29	0.0222 (6)	0.0206 (6)	0.0224 (6)	−0.0065 (5)	0.0068 (5)	−0.0005 (5)
C30	0.0154 (5)	0.0247 (6)	0.0259 (6)	−0.0018 (5)	0.0069 (5)	0.0029 (5)
C31	0.0175 (5)	0.0173 (5)	0.0242 (6)	0.0007 (4)	0.0093 (5)	0.0020 (5)
C32	0.0143 (5)	0.0176 (5)	0.0206 (5)	0.0009 (4)	0.0089 (4)	−0.0020 (4)
C33	0.0217 (6)	0.0169 (5)	0.0223 (6)	−0.0015 (5)	0.0088 (5)	−0.0005 (4)
C34	0.0299 (7)	0.0217 (6)	0.0225 (6)	−0.0003 (5)	0.0085 (5)	0.0020 (5)
C35	0.0298 (7)	0.0170 (6)	0.0336 (7)	0.0010 (5)	0.0162 (6)	0.0039 (5)
C36	0.0211 (6)	0.0153 (5)	0.0371 (7)	−0.0020 (5)	0.0145 (5)	−0.0051 (5)
C37	0.0169 (5)	0.0199 (6)	0.0263 (6)	0.0005 (5)	0.0075 (5)	−0.0044 (5)
C38	0.0195 (5)	0.0177 (5)	0.0196 (5)	0.0061 (4)	0.0080 (4)	0.0018 (4)
C39	0.0248 (6)	0.0198 (6)	0.0255 (6)	0.0063 (5)	0.0124 (5)	0.0039 (5)
C40	0.0383 (8)	0.0256 (7)	0.0337 (7)	0.0118 (6)	0.0239 (6)	0.0081 (6)
C41	0.0489 (9)	0.0290 (7)	0.0235 (6)	0.0183 (6)	0.0196 (6)	0.0044 (5)
C42	0.0385 (8)	0.0314 (7)	0.0212 (6)	0.0101 (6)	0.0071 (6)	−0.0027 (5)
C43	0.0248 (6)	0.0271 (6)	0.0224 (6)	0.0040 (5)	0.0082 (5)	−0.0019 (5)

### Geometric parameters (Å, º)

**Table:**

B1—C20	1.6483 (17)	C20—C21	1.4022 (17)
B1—C26	1.6483 (18)	C20—C25	1.4054 (17)
B1—C38	1.6487 (17)	C21—C22	1.3931 (17)
B1—C32	1.6529 (18)	C21—H21A	0.9500
N1—N2	1.3132 (14)	C22—C23	1.387 (2)
N1—C1	1.3474 (15)	C22—H22A	0.9500
N2—N3	1.3392 (14)	C23—C24	1.383 (2)
N2—C8	1.4452 (15)	C23—H23A	0.9500
N3—N4	1.3118 (14)	C24—C25	1.3962 (17)
N3—C14	1.4452 (15)	C24—H24A	0.9500
N4—C1	1.3468 (16)	C25—H25A	0.9500
C1—C2	1.4631 (16)	C26—C27	1.4036 (16)
C2—C7	1.3930 (18)	C26—C31	1.4117 (16)
C2—C3	1.3977 (18)	C27—C28	1.3911 (17)
C3—C4	1.3880 (18)	C27—H27A	0.9500
C3—H3A	0.9500	C28—C29	1.3898 (17)
C4—C5	1.385 (2)	C28—H28A	0.9500
C4—H4A	0.9500	C29—C30	1.3878 (18)
C5—C6	1.384 (2)	C29—H29A	0.9500
C5—H5A	0.9500	C30—C31	1.3903 (17)
C6—C7	1.3889 (18)	C30—H30A	0.9500
C6—H6A	0.9500	C31—H31A	0.9500
C7—H7A	0.9500	C32—C33	1.4027 (16)
C8—C9	1.3853 (17)	C32—C37	1.4083 (17)
C8—C13	1.3862 (17)	C33—C34	1.3928 (17)
C9—C10	1.3908 (18)	C33—H33A	0.9500
C9—H9A	0.9500	C34—C35	1.3828 (18)
C10—C11	1.3841 (19)	C34—H34A	0.9500
C10—H10A	0.9500	C35—C36	1.3893 (19)
C11—C12	1.391 (2)	C35—H35A	0.9500
C11—H11A	0.9500	C36—C37	1.3915 (18)
C12—C13	1.3852 (18)	C36—H36A	0.9500
C12—H12A	0.9500	C37—H37A	0.9500
C13—H13A	0.9500	C38—C39	1.4024 (17)
C14—C19	1.3828 (17)	C38—C43	1.4068 (18)
C14—C15	1.3860 (17)	C39—C40	1.3971 (18)
C15—C16	1.3889 (18)	C39—H39A	0.9500
C15—H15A	0.9500	C40—C41	1.379 (2)
C16—C17	1.387 (2)	C40—H40A	0.9500
C16—H16A	0.9500	C41—C42	1.386 (2)
C17—C18	1.3854 (19)	C41—H41A	0.9500
C17—H17A	0.9500	C42—C43	1.3953 (18)
C18—C19	1.3880 (18)	C42—H42A	0.9500
C18—H18A	0.9500	C43—H43A	0.9500
C19—H19A	0.9500		
			
C20—B1—C26	107.39 (9)	C21—C20—B1	122.26 (10)
C20—B1—C38	110.02 (9)	C25—C20—B1	122.10 (10)
C26—B1—C38	111.79 (9)	C22—C21—C20	122.85 (12)
C20—B1—C32	107.10 (9)	C22—C21—H21A	118.6
C26—B1—C32	109.40 (9)	C20—C21—H21A	118.6
C38—B1—C32	110.96 (9)	C23—C22—C21	120.14 (13)
N2—N1—C1	103.80 (10)	C23—C22—H22A	119.9
N1—N2—N3	109.81 (9)	C21—C22—H22A	119.9
N1—N2—C8	124.79 (10)	C24—C23—C22	118.90 (12)
N3—N2—C8	125.24 (10)	C24—C23—H23A	120.6
N4—N3—N2	110.31 (9)	C22—C23—H23A	120.6
N4—N3—C14	123.29 (10)	C23—C24—C25	120.39 (12)
N2—N3—C14	126.37 (10)	C23—C24—H24A	119.8
N3—N4—C1	103.59 (10)	C25—C24—H24A	119.8
N4—C1—N1	112.48 (10)	C24—C25—C20	122.45 (12)
N4—C1—C2	122.61 (11)	C24—C25—H25A	118.8
N1—C1—C2	124.92 (11)	C20—C25—H25A	118.8
C7—C2—C3	120.54 (11)	C27—C26—C31	114.55 (11)
C7—C2—C1	120.31 (11)	C27—C26—B1	122.82 (10)
C3—C2—C1	119.14 (11)	C31—C26—B1	122.51 (10)
C4—C3—C2	119.29 (13)	C28—C27—C26	123.02 (11)
C4—C3—H3A	120.4	C28—C27—H27A	118.5
C2—C3—H3A	120.4	C26—C27—H27A	118.5
C5—C4—C3	120.14 (13)	C29—C28—C27	120.44 (12)
C5—C4—H4A	119.9	C29—C28—H28A	119.8
C3—C4—H4A	119.9	C27—C28—H28A	119.8
C6—C5—C4	120.52 (12)	C30—C29—C28	118.59 (11)
C6—C5—H5A	119.7	C30—C29—H29A	120.7
C4—C5—H5A	119.7	C28—C29—H29A	120.7
C5—C6—C7	120.11 (13)	C29—C30—C31	120.14 (11)
C5—C6—H6A	119.9	C29—C30—H30A	119.9
C7—C6—H6A	119.9	C31—C30—H30A	119.9
C6—C7—C2	119.40 (13)	C30—C31—C26	123.20 (11)
C6—C7—H7A	120.3	C30—C31—H31A	118.4
C2—C7—H7A	120.3	C26—C31—H31A	118.4
C9—C8—C13	123.73 (11)	C33—C32—C37	114.85 (11)
C9—C8—N2	117.85 (11)	C33—C32—B1	122.48 (10)
C13—C8—N2	118.41 (11)	C37—C32—B1	122.07 (10)
C8—C9—C10	117.37 (12)	C34—C33—C32	122.95 (12)
C8—C9—H9A	121.3	C34—C33—H33A	118.5
C10—C9—H9A	121.3	C32—C33—H33A	118.5
C11—C10—C9	120.36 (12)	C35—C34—C33	120.50 (12)
C11—C10—H10A	119.8	C35—C34—H34A	119.7
C9—C10—H10A	119.8	C33—C34—H34A	119.7
C10—C11—C12	120.72 (12)	C34—C35—C36	118.41 (12)
C10—C11—H11A	119.6	C34—C35—H35A	120.8
C12—C11—H11A	119.6	C36—C35—H35A	120.8
C13—C12—C11	120.25 (12)	C35—C36—C37	120.54 (12)
C13—C12—H12A	119.9	C35—C36—H36A	119.7
C11—C12—H12A	119.9	C37—C36—H36A	119.7
C12—C13—C8	117.57 (12)	C36—C37—C32	122.67 (12)
C12—C13—H13A	121.2	C36—C37—H37A	118.7
C8—C13—H13A	121.2	C32—C37—H37A	118.7
C19—C14—C15	123.73 (11)	C39—C38—C43	114.71 (11)
C19—C14—N3	117.29 (11)	C39—C38—B1	124.08 (11)
C15—C14—N3	118.95 (11)	C43—C38—B1	121.21 (11)
C14—C15—C16	117.35 (12)	C40—C39—C38	123.01 (13)
C14—C15—H15A	121.3	C40—C39—H39A	118.5
C16—C15—H15A	121.3	C38—C39—H39A	118.5
C17—C16—C15	120.47 (12)	C41—C40—C39	120.28 (13)
C17—C16—H16A	119.8	C41—C40—H40A	119.9
C15—C16—H16A	119.8	C39—C40—H40A	119.9
C18—C17—C16	120.43 (12)	C40—C41—C42	118.92 (12)
C18—C17—H17A	119.8	C40—C41—H41A	120.5
C16—C17—H17A	119.8	C42—C41—H41A	120.5
C17—C18—C19	120.54 (13)	C41—C42—C43	120.14 (13)
C17—C18—H18A	119.7	C41—C42—H42A	119.9
C19—C18—H18A	119.7	C43—C42—H42A	119.9
C14—C19—C18	117.45 (12)	C42—C43—C38	122.93 (13)
C14—C19—H19A	121.3	C42—C43—H43A	118.5
C18—C19—H19A	121.3	C38—C43—H43A	118.5
C21—C20—C25	115.27 (11)		
			
C1—N1—N2—N3	0.55 (12)	C38—B1—C20—C25	149.98 (11)
C1—N1—N2—C8	−175.09 (11)	C32—B1—C20—C25	29.28 (15)
N1—N2—N3—N4	−1.01 (13)	C25—C20—C21—C22	−0.38 (18)
C8—N2—N3—N4	174.60 (10)	B1—C20—C21—C22	−173.49 (11)
N1—N2—N3—C14	177.07 (10)	C20—C21—C22—C23	0.3 (2)
C8—N2—N3—C14	−7.32 (18)	C21—C22—C23—C24	0.2 (2)
N2—N3—N4—C1	0.99 (13)	C22—C23—C24—C25	−0.6 (2)
C14—N3—N4—C1	−177.16 (11)	C23—C24—C25—C20	0.5 (2)
N3—N4—C1—N1	−0.66 (13)	C21—C20—C25—C24	0.01 (18)
N3—N4—C1—C2	179.49 (11)	B1—C20—C25—C24	173.13 (11)
N2—N1—C1—N4	0.07 (13)	C20—B1—C26—C27	−3.63 (15)
N2—N1—C1—C2	179.92 (11)	C38—B1—C26—C27	117.14 (12)
N4—C1—C2—C7	−176.73 (12)	C32—B1—C26—C27	−119.54 (12)
N1—C1—C2—C7	3.44 (19)	C20—B1—C26—C31	172.03 (10)
N4—C1—C2—C3	2.08 (18)	C38—B1—C26—C31	−67.20 (14)
N1—C1—C2—C3	−177.75 (12)	C32—B1—C26—C31	56.12 (14)
C7—C2—C3—C4	−0.4 (2)	C31—C26—C27—C28	−2.43 (17)
C1—C2—C3—C4	−179.25 (12)	B1—C26—C27—C28	173.54 (11)
C2—C3—C4—C5	0.1 (2)	C26—C27—C28—C29	0.57 (19)
C3—C4—C5—C6	0.4 (2)	C27—C28—C29—C30	1.63 (18)
C4—C5—C6—C7	−0.5 (2)	C28—C29—C30—C31	−1.80 (18)
C5—C6—C7—C2	0.1 (2)	C29—C30—C31—C26	−0.22 (19)
C3—C2—C7—C6	0.34 (19)	C27—C26—C31—C30	2.26 (17)
C1—C2—C7—C6	179.13 (12)	B1—C26—C31—C30	−173.72 (11)
N1—N2—C8—C9	−53.50 (16)	C20—B1—C32—C33	−105.11 (12)
N3—N2—C8—C9	131.52 (12)	C26—B1—C32—C33	10.99 (15)
N1—N2—C8—C13	125.11 (13)	C38—B1—C32—C33	134.80 (11)
N3—N2—C8—C13	−49.87 (16)	C20—B1—C32—C37	65.54 (13)
C13—C8—C9—C10	0.15 (19)	C26—B1—C32—C37	−178.36 (10)
N2—C8—C9—C10	178.68 (11)	C38—B1—C32—C37	−54.56 (14)
C8—C9—C10—C11	0.09 (18)	C37—C32—C33—C34	−1.34 (17)
C9—C10—C11—C12	0.0 (2)	B1—C32—C33—C34	169.93 (11)
C10—C11—C12—C13	−0.3 (2)	C32—C33—C34—C35	−1.2 (2)
C11—C12—C13—C8	0.5 (2)	C33—C34—C35—C36	2.3 (2)
C9—C8—C13—C12	−0.44 (19)	C34—C35—C36—C37	−0.83 (19)
N2—C8—C13—C12	−178.96 (11)	C35—C36—C37—C32	−1.84 (19)
N4—N3—C14—C19	−51.05 (16)	C33—C32—C37—C36	2.83 (17)
N2—N3—C14—C19	131.10 (13)	B1—C32—C37—C36	−168.48 (11)
N4—N3—C14—C15	126.99 (13)	C20—B1—C38—C39	137.58 (11)
N2—N3—C14—C15	−50.86 (17)	C26—B1—C38—C39	18.35 (16)
C19—C14—C15—C16	−1.55 (19)	C32—B1—C38—C39	−104.08 (13)
N3—C14—C15—C16	−179.46 (11)	C20—B1—C38—C43	−41.86 (15)
C14—C15—C16—C17	−0.1 (2)	C26—B1—C38—C43	−161.09 (11)
C15—C16—C17—C18	1.5 (2)	C32—B1—C38—C43	76.48 (13)
C16—C17—C18—C19	−1.4 (2)	C43—C38—C39—C40	0.58 (18)
C15—C14—C19—C18	1.7 (2)	B1—C38—C39—C40	−178.89 (11)
N3—C14—C19—C18	179.61 (11)	C38—C39—C40—C41	−0.2 (2)
C17—C18—C19—C14	−0.2 (2)	C39—C40—C41—C42	−0.2 (2)
C26—B1—C20—C21	84.50 (13)	C40—C41—C42—C43	0.3 (2)
C38—B1—C20—C21	−37.38 (15)	C41—C42—C43—C38	0.1 (2)
C32—B1—C20—C21	−158.07 (11)	C39—C38—C43—C42	−0.49 (18)
C26—B1—C20—C25	−88.14 (13)	B1—C38—C43—C42	179.00 (12)

### Hydrogen-bond geometry (Å, º)

*Cg*1 is the centroid of the C38–C43 ring.

**Table:**

*D*—H···*A*	*D*—H	H···*A*	*D*···*A*	*D*—H···*A*
C10—H10*A*···*Cg*1^i^	0.95	2.93	3.7784 (15)	149

Symmetry code: (i) −*x*+2, −*y*+2, −*z*+1.
